# Multiparametric cardiac magnetic resonance imaging in pediatric and adolescent patients with acute myocarditis

**DOI:** 10.1007/s00247-021-05169-7

**Published:** 2021-08-25

**Authors:** Alexander Isaak, Leon M. Bischoff, Anton Faron, Christoph Endler, Narine Mesropyan, Alois M. Sprinkart, Claus C. Pieper, Daniel Kuetting, Darius Dabir, Ulrike Attenberger, Julian A. Luetkens

**Affiliations:** 1 Department of Diagnostic and Interventional Radiology, University Hospital Bonn, Venusberg-Campus 1, 53127 Bonn, Germany; 2 Quantitative Imaging Lab Bonn (QILaB), University Hospital Bonn, Bonn, Germany

**Keywords:** Adolescents, Cardiac magnetic resonance imaging, Children, Heart, Lake Louise criteria, Myocarditis, T1 mapping, T2 mapping, Young adults

## Abstract

**Background:**

The diagnostic value of cardiac magnetic resonance imaging (MRI) employing the 2018 Lake Louise criteria in pediatric and adolescent patients with acute myocarditis is undefined.

**Objective:**

To evaluate the diagnostic value of the Lake Louise criteria in pediatric and adolescent patients with suspected acute myocarditis and to show the utility of cardiac MRI for follow-up in this patient cohort.

**Materials and methods:**

Forty-three patients (age range: 8–21 years) with suspected acute myocarditis and 13 control patients who underwent cardiac MRI were retrospectively analyzed. T2-weighted and late gadolinium enhancement imaging were performed in all patients. T1 and T2 mapping were available in 26/43 patients (60%). The Lake Louise criteria were assessed. In 27/43 patients (63%), cardiac MRI follow-up was available. Receiver operating characteristic analysis, Pearson’s correlation coefficient and paired Student’s *t*-test were used for statistical analysis.

**Results:**

In the total cohort, the Lake Louise criteria achieved a sensitivity of 86% (95% confidence interval [CI]: 72–95%) and a specificity of 100% (95% CI: 79–100%) for the diagnosis of acute myocarditis. In the subgroup of patients with available mapping parameters, the diagnostic performance of the Lake Louise criteria was higher when mapping parameters were implemented into the score (area under the receiver operating characteristic curve: 0.944 vs. 0.870; *P*=0.033). T2 relaxation times were higher in patients with admission to the intermediate care unit and were associated with the length of intermediate care unit stay (r=0.879, *P*=0.049). Cardiac MRI markers of active inflammation decreased on follow-up examinations (e.g., T1 relaxation times: 1,032±39 ms vs. 975±33 ms, *P*<0.001; T2 relaxation times: 58±5 ms vs. 54±5 ms, *P*=0.003).

**Conclusion:**

The Lake Louise criteria have a high diagnostic performance for the diagnosis of acute myocarditis and are a valuable tool for follow-up in pediatric and adolescent patients. The mapping techniques enhance the diagnostic performance of the 2018 Lake Louise criteria.

**Supplementary Information:**

The online version contains supplementary material available at 10.1007/s00247-021-05169-7.

## Introduction

Acute myocarditis is an inflammatory disorder of the myocardium primarily caused by infections, autoimmune processes, systemic diseases, drugs or toxins and can affect both adults and children [1]. Because subclinical manifestation is common, the true incidence of acute myocarditis in children is unknown. Estimated incidence rates range between 1 and 2 per 100,000 children [2, 3]. Males seem to be affected more often [2, 4]. Although incidence of acute myocarditis is relatively low in children, it accounts for about one-third of childhood dilated cardiomyopathies [5] and is related to sudden cardiac death in young patients [6, 7]. Diagnosis of myocarditis is challenging because of the wide spectrum of clinical presentation. Furthermore, children particularly may show atypical manifestations [8]. The clinical course is heterogeneous, varying from fast convalescence to delayed recovery or chronic heart failure and, rarely, serious long-term morbidity [8, 9]. Due to its invasiveness and possible sampling error, endomyocardial biopsy — the diagnostic reference standard — is used infrequently in clinical practice. Nowadays, cardiac magnetic resonance imaging (MRI) plays an important role for diagnostic work-up in patients with suggested myocarditis. Cardiac MRI is a noninvasive and radiation-free technique that is suitable for pediatric and adolescent patients and can be of particular value to differentiate acute myocarditis from myocardial infarction, primary cardiomyopathy or congenital heart disease to spare young patients from coronary angiography [10, 11]. The Lake Louise criteria were established in 2009 for the diagnosis of myocarditis by cardiac MRI. They include three aspects of myocardial inflammation (edema, hyperemia and necrosis) in a two-out-of-three approach (high signal intensities on T2-weighted images, early gadolinium enhancement and late gadolinium enhancement) [12]. In 2018, the implementation of quantitative mapping techniques led to a revision of the original Lake Louise criteria [13–15]. According to the 2018 Lake Louise criteria, diagnosis of myocarditis can be made when two main criteria are fulfilled, at least one T1-based criterion (increased myocardial T1 relaxation time, increased extracellular volume fraction or positive late gadolinium enhancement) and one T2-based criterion (increased myocardial T2 relaxation time or visual myocardial edema/increased T2 signal intensity ratio) [16]. Previous studies evaluated the performance of cardiac MRI diagnostic parameters primary in adults, but the diagnostic value of quantitative parameters remains undetermined in children and adolescents.

The purpose of this cardiac MRI study was to evaluate (1) the diagnostic performance of the 2018 Lake Louise criteria for acute myocarditis in pediatric and adolescent patients and (2) to assess the value of cardiac MRI for follow-up of the disease.

## Materials and methods

This retrospective, case-control study was approved by the institutional ethics committee and the requirement for written informed consent was waived. Patients with clinically defined acute myocarditis and control patients were included in this study. The department’s registry was searched for children and adolescents (ages 2 to 21 years old, according to the American Academy of Pediatrics [17]) who had undergone cardiac MRI for the evaluation of acute myocarditis on the same clinical whole-body MRI system between January 2014 and April 2020. Patients with preexisting cardiovascular disease were excluded. The diagnosis of myocarditis was made according to current criteria for clinically suspected myocarditis as recommended by the European Society of Cardiology Working Group on Myocardial and Pericardial Diseases [8]. The main criteria for clinical diagnosis were acute chest pain, signs of acute myocardial injury (electrocardiogram [ECG] changes and/or elevated troponin), and a constellation of signs associated with infection (elevated leucocytes and/or C-reactive protein and/or confirmed infectious disease). This clinical validation approach was used for the diagnosis of acute myocarditis in this study as previously reported [14–16, 18]. Clinical evidence was the reference standard against which the diagnostic performance of cardiac MRI parameters was tested. As a control group, outpatients were retrospectively reviewed for those who had been referred for nonspecific cardiac symptoms or thoracic discomfort to rule out structural heart disease. All control patients had an unremarkable past medical history of cardiovascular disease. Electrocardiographic and echocardiographic results were unremarkable and no cardiac risk factors were present. All control participants had normal cardiac MRI results without structural abnormalities.

### Cardiac magnetic resonance

All investigations were performed on a clinical whole-body MRI system (Ingenia 1.5 tesla; Philips Healthcare, Best, the Netherlands). For signal reception, a 32-channel torso coil with digital interface was used. ECG-gated steady state free precession cine images were acquired in short-axis, four-chamber and two-chamber views. T2-weighted short tau inversion recovery (STIR) sequences were acquired in short-axis, two-chamber and transversal views to visualize myocardial edema and calculate the T2 signal intensity ratio. Late gadolinium enhancement imaging was based on segmented inversion recovery gradient echo sequences in short-axis, two-chamber and four-chamber orientation. Cardiac MRI protocol consisted of myocardial T1 and T2 mapping acquired in end-diastole in apical, midventricular and basal short-axis views. Post-contrast myocardial T1 maps were performed 10 min after contrast injection in the same orientation. T1 mapping was based on a standard 3(3)3(3)5 modified Look-Locker inversion recovery (MOLLI) acquisition scheme [19]. A 6-echo gradient spin echo (GraSE) sequence allowed acquisition of T2 mapping [20]. For contrast enhancement, a single bolus of 0.2 mmol/kg body weight of gadobutrol (Gadovist; Bayer Healthcare, Berlin, Germany) was given. For extracellular volume fraction estimation, the hematocrit blood levels on the cardiac MRI day were available. A detailed description of the cardiac MRI sequence parameters is provided in Online Supplementary Material 1.

### Cardiac image analysis

Images were analyzed by two radiologists (J.A.L. and A.I., with 8 and 3 years of experience in cardiac MRI, respectively) using dedicated software (IntelliSpace Portal version 10.1; Philips Medical System, Best, the Netherlands). Readers were blinded to the clinical information. All volumes and masses were indexed to body surface area using the Mosteller method. Visual analysis of T2 STIR and late gadolinium enhancement images was evaluated separately by consensus agreement of the two readers for focal myocardial hyperintensity as markers for myocardial edema and necrosis, respectively. Semiquantitative analysis allowed for determination of quantitative T2 signal intensity ratio (global myocardial edema) [12, 14]. Myocardial relaxation maps were motion corrected using FEIR (fast elastic image registration) by dedicated software (IntelliSpace Portal version 10.1, Philips Medical System). Myocardial T1 and T2 relaxation times and hematocrit corrected extracellular volume fraction values (using pre- and post-contrast T1 values) were calculated using a segmental approach as previously described [14–16]. The 2018 Lake Louise criteria were applied as recommended [13].

### Statistical analysis

Prism (version 8.4.3; GraphPad Software, San Diego, CA), SPSS Statistics (version 26; IBM Corp., Armonk, NY) and MedCalc (version 18.11.3; MedCalc Software bvba, Ostend, Belgium) were used for statistical analysis. The Kolmogorov-Smirnov test was applied to assess normality. Continuous patient characteristics are presented as mean±standard deviation or as absolute frequency. Continuous variables between the two groups were compared using the Student’s *t*-test. Dichotomous variables were compared using the Fisher exact test. For intraindividual comparisons, the paired Student’s *t*-test and McNemar’s test were used. Correlation analysis was performed using Pearson’s correlation coefficient. A receiver operating characteristic (ROC) analysis was performed to calculate area under the curve (AUC). Optimal cutoff values were determined using the Youden index and sensitivities and specificities were calculated. Differences between ROC curves were tested using the DeLong method. The level of statistical significance was set to *P*<0.05.

## Results

### General characteristics

Fifty-nine subjects, 43 patients with acute myocarditis (77% males, mean age: 17±3 years, range: 8–21 years) and 16 age-matched control patients (50% males, mean age: 17±4 years, range: 5–21 years) were included in this study (Table 1). Cardiac MRI was performed 4±5 days after clinical diagnosis of suspected myocarditis. Acute thoracic pain was present in 26/43 (60%), abnormal ECG in 35/43 (81%) and infectious disease in 31/43 (72%) patients. Coronary angiography was performed in 11/43 patients (26%) to rule out coronary stenosis. Patients had significantly elevated blood markers of troponin, white blood cell count and C-reactive protein when compared to healthy controls (Table 1). Nine of the 43 (21%) patients with acute myocarditis were admitted to the intensive care unit (length of stay: 7±8 days) during their hospital stay.

**Table 1:** Clinical and cardiac magnetic resonance imaging (MRI) characteristics of pediatric and adolescent patients with acute myocarditis and control patients

Variable	Patients (*n*=43)	Controls (*n*=16)	*P*-value
Clinical parameters			
Age (years)	17±3	17±4	0.35
Men	33 (77%)	8 (50%)	0.061
Weight (kg)	72±19	61±20	0.068
Height (cm)	168±17	160±16	0.10
Heart rate (bpm)	73±15	76±10	0.46
Hematocrit (%)	39±6	39±3	0.67
Elevated troponin	40 (93%)	0 (0%)	<0.001
White blood cell count (10^3^/μL)	10.8±5.0	8.6±1.6	0.021
C-reactive protein (mg/L)	62±1	2±2	<0.001
General cardiac MRI parameters			
Left ventricular ejection fraction (%)	57±8	59±5	0.32
Left ventricular end-diastolic volume index (mL/m^2^)	83±15	78±12	0.17
Cardiac index (L/min/m^2^)	3.3±0.6	3.4±0.5	0.72
Interventricular septal thickness (mm)	8.3±1.4	7.5±1.4	0.20
Pericardial effusion	17 (40%)	0 (0%)	<0.001
T2 signal intensity ratio	2.10±0.49	1.54±0.29	<0.001
Visual focal myocardial edema	32 (74%)	0 (0%)	<0.001
Visual late gadolinium enhancement	36 (84%)	0 (0%)	<0.001
Mapping cardiac MRI parameters			
T1 relaxation time, native (ms)	1,031±46	962±17	<0.001
Extracellular volume fraction (%)	29.2±5.9	26.5±2.8	0.058
T2 relaxation time (ms)	58±5	51±2	<0.001

Continuous variables are given as mean±standard deviation. Dichotomous variables are given as absolute frequency with percentages in parentheses. *P*-values were obtained using a Student’s *t*-test, χ2 test (cell count >5) or Fisher exact test (cell count ≤5). T1 and T2 relaxation times and extracellular volume fraction were available in 26/43 patients and all controls

### Cardiac magnetic resonance imaging results

No significant differences were observed in left ventricular ejection fraction (LVEF; 57±8% vs. 59±5%; *P*=0.32), left ventricular end-diastolic volume index (83±15 mL/m^2^ vs. 78±12 mL/m^2^; *P*=0.17), or cardiac index (3.3±0.6 L/min/m^2^ vs. 3.4±0.5 L/min/m^2^; *P*=0.72) between patients with acute myocarditis and control patients (Table 1). Twenty-nine of the 43 (67%) myocarditis patients had preserved LVEF ≥55%. Pericardial effusion was present in 17/43 (40%) patients (controls: 0/16, 0%; *P*<0.001). Focal high intensities on T2 STIR images were detected in 32/43 (74%) patients with acute myocarditis (controls: 0/16, 0%; *P*<0.001), and T2 signal intensity ratio was elevated in the myocarditis group compared to the control group (2.10±0.49 vs. 1.54±0.29, *P*<0.001). Late gadolinium enhancement in a nonischemic distribution was found in 36/43 (84%) patients (controls: 0/16, 0%; *P*<0.001). Positive late gadolinium enhancement was observed more frequently in patients with reduced LVEF (<55%) than in patients with preserved LVEF (≥55%) (14/14, 100% vs. 22/29, 76%; *P*=0.048).

**Fig. 1:**
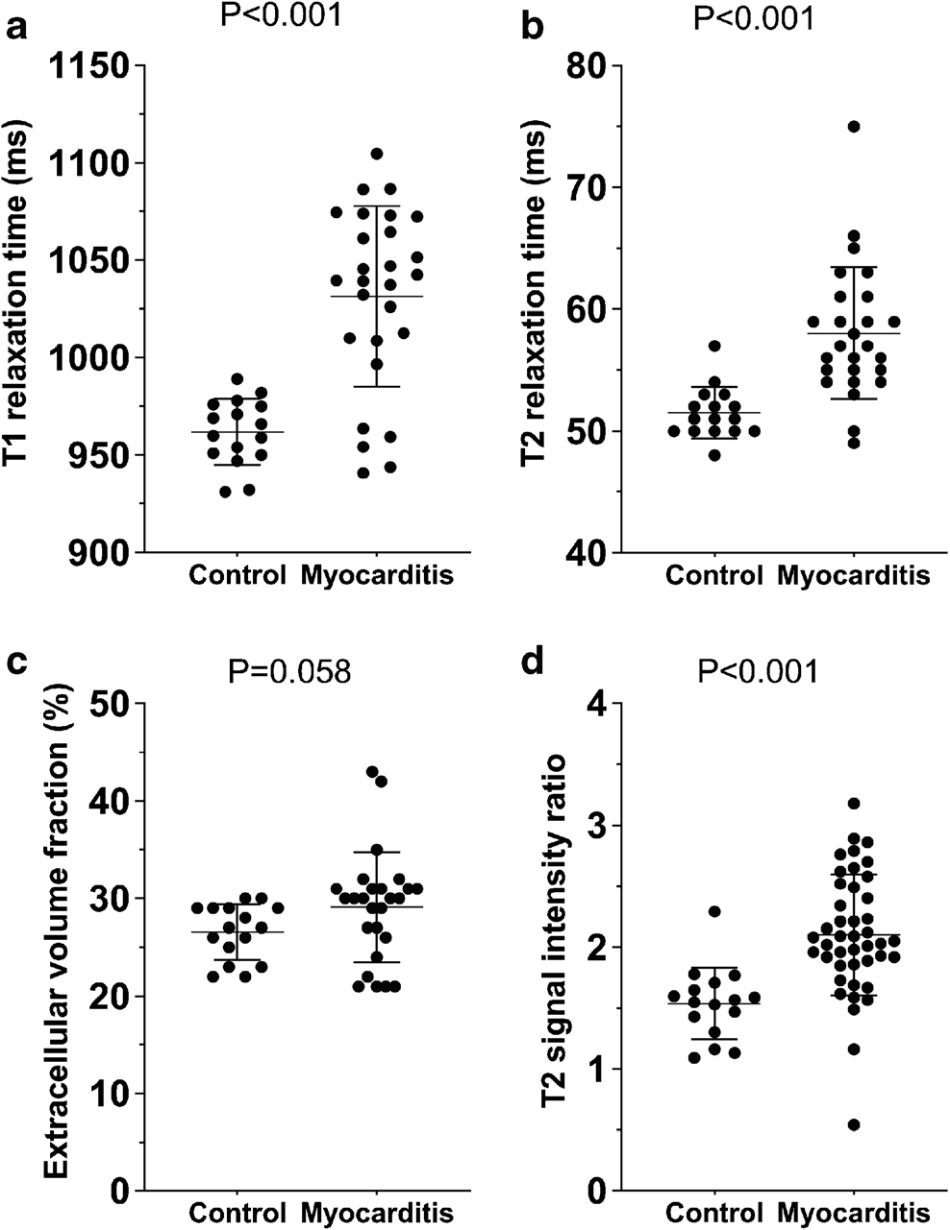
Graphs with individual plotted values show the distribution of myocardial magnetic resonance parameters in controls and in the subgroup of patients with myocarditis and available mapping parameters (26/43 patients and 16/16 controls). **a** T1 relaxation time. **b** T2 relaxation time. **c** Extracellular volume fraction. **d** T2 signal intensity ratio. T2 signal intensity ratio was available in all patients and controls. Individual values are represented as single *dots*. The *horizontal lines* show the mean values with error bars representing one standard deviation. *P*-values were obtained using an unpaired Student’s *t*-test

T1 and T2 mapping was available in a subgroup of 26/43 patients and in all control patients. Myocardial native T1 relaxation time as a marker of acute myocardial injury was higher in patients with acute myocarditis than in healthy controls (1,031±46 ms vs. 962±17 ms; *P*<0.001) (Fig. 1).

T2 relaxation time, as a marker of diffuse myocardial edema, was higher in patients compared to controls (58±5 ms vs. 51±2 ms; *P*<0.001). No difference was observed in extracellular volume fraction values between both groups (29.2±5.9% vs. 26.5±2.8%; *P*=0.058). Quantitative parameters of myocardial inflammation were higher in patients with reduced LVEF (T1 relaxation time: 1,066±17 ms vs. 1,008±58 ms, *P*=0.001; T2 relaxation time: 63±6 ms vs. 56±4 ms, *P*=0.010). Patients with admission to the intermediate care unit had more elevated T1 (1,063±26 ms vs. 1,017±58 ms; *P*=0.011) and T2 relaxation times (64±6 ms vs. 56±4 ms; *P*=0.015), but no significant differences in extracellular volume fraction (32.1±5.8% vs. 26.1±5.5%; *P*=0.057), LVEF (51±11% vs. 59±6%; *P*=0.059) or troponin level (787±702 ng/L vs. 739±783 ng/L; *P*=0.90) when compared to patients without admission to the intermediate care unit. T2 relaxation time as a parameter of myocardial edema was associated with the length of intermediate care unit stay (r=0.879, *P*=0.049) and inflammatory laboratory parameters (C-reactive protein: r=0.532, *P*=0.009; white blood cell count: r=0.589, *P*=0.002). T1 relaxation times correlated with troponin levels (r=0.558, *P*=0.016). Patients younger than 18 years old displayed lower parameters of myocardial edema (T2 relaxation time: 55±3 ms vs. 59±6 ms, *P*=0.035; T2 signal intensity ratio: 1.88±0.34 vs 2.23±0.53, *P*=0.012), but no difference was found in T1 relaxation time (1,035±40 ms vs. 1,024±61 ms; *P*=0.59) when compared to myocarditis patients ages ≥18 years.

### Diagnostic performance

In the total cohort (*n*=43), the 2018 Lake Louise criteria could only be applied in a limited way because mapping parameters were not available in 17/43 patients. The application of the 2018 Lake Louise criteria in the total cohort reached a sensitivity of 37/43 (86%, 95% confidence interval [CI]: 72–95%) and a specificity of 16/16 (100%, 95% CI: 79–100%) with an AUC of 0.930. Single parameters alone showed lower sensitivity (late gadolinium enhancement: 36/43 [84%, 95% CI: 69–93%], visual focal edema: 32/43 [74%, 95% CI: 59–87%], T2 signal intensity ratio: 34/43 [79%, 95% CI: 64–90%]).

In the subgroup analysis of patients with available mapping parameters (*n*=26/43), high diagnostic performance was achieved by T2 mapping with an AUC of 0.899 (sensitivity: 23/26 [88%, 95% CI: 70–98%], specificity: 13/16 [88%, 95% CI: 62–98%]), by T1 mapping with an AUC of 0.873 (sensitivity: 21/26 [82%, 95% CI: 62–94%], specificity: 16/16 [100%, 95% CI: 79–100%]), and by T2 signal intensity ratio with an AUC of 0.868 (sensitivity: 20/26, [77%, 95% CI: 64–90%], specificity: 15/16 [94%, 95% CI: 70–100%]). Extracellular volume fraction had an AUC of 0.687 (sensitivity: 15/26 [58%, 95% CI: 37–77%], specificity: 14/16 [88%, 95% CI: 62–98%]). In this subgroup, the 2018 Lake Louise criteria yielded a significantly higher diagnostic performance (AUC: 0.944, sensitivity: 89%, specificity: 100%) compared to the modified 2018 Lake Louise criteria with exclusion of mapping parameters (AUC: 0.870, sensitivity: 73%, specificity: 100%; *P*=0.034). Mapping parameters allowed for the diagnosis of acute myocarditis in four additional patients. Furthermore, a combined non-contrast score of native T1 and T2 relaxation times yielded an AUC of 0.920 and a sensitivity of 22/26 (85%, 95% CI: 64–96%) and a specificity of 16/16 (100%, 95% CI: 79–100%). Comparison of the ROC curves of this non-contrast score and the 2018 Lake Louise criteria showed no significant difference in diagnostic performance (*P*=0.15).

**Fig. 2:**
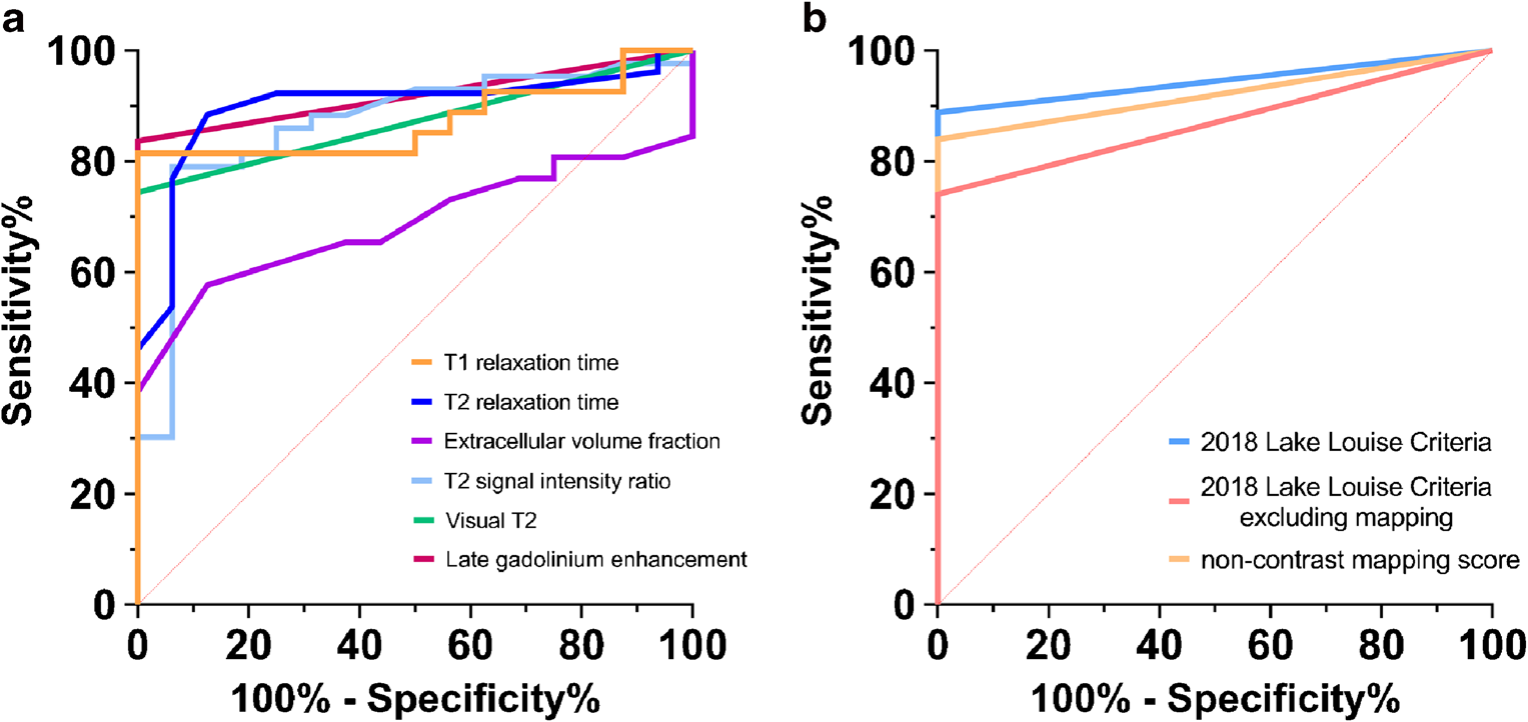
Graphs show receiver operating characteristic (ROC) curves for the subgroup cohort of patients (26/43) and control patients (16/16) with available mapping parameters. **a** The individual performance of different cardiac magnetic resonance parameters are presented: native T1 relaxation time (area under the curve [AUC]: 0.873), T2 relaxation time (AUC: 0.899), extracellular volume (AUC: 0.687), T2 signal intensity ratio (AUC: 0.868) and late gadolinium enhancement (AUC: 0.919). **b** The performance of combined scores is visualized: 2018 Lake Louise criteria (AUC: 0.944), 2018 Lake Louise criteria excluding mapping parameters (AUC: 0.870), and a non-contrast score of native T1 and T2 relaxation times only (AUC: 0.920)

Table 2 summarizes all cutoff values, sensitivities and specificities with confidence intervals for all evaluated parameters. Figure 2 visualizes the AUC values for single variables and the 2018 Lake Louise criteria of the total cohort, and a combined score of native quantitative myocardial parameters.

**Table 2:** Diagnostic performance of single and combined cardiac magnetic resonance imaging parameters for diagnosis of acute myocarditis in pediatric and adolescent patients

Variable	AUC	Cutoff	Sensitivity (%)	Specificity (%)
Qualitative/semiquantitative parameters				
Visual late gadolinium enhancement	0.919 (0.818–0.974)		84 (69–93)	100 (79–100)
Visual focal myocardial edema	0.872 (0.759–0.945)		74 (59–87)	100 (79–100)
T2 signal intensity ratio	0.868 (0.755–0.942)	>1.78	79 (64–90)	94 (70–100)
Quantitative parameters				
T1 relaxation time	0.873 (0.736–0.955)	>993 ms	82 (62–94)	100 (79–100)
Extracellular volume fraction	0.687 (0.526–0.821)	>29%	58 (37–77)	88 (62–98)
T2 relaxation time	0.899 (0.766–0.970)	>53 ms	88 (70–98)	88 (62–98)
Combinations (total cohort)				
2018 Lake Louise criteria	0.930 (0.833–0.980)		86 (72–95)	100 (79–100)
Combinations (subgroup cohort^a^)				
2018 Lake Louise criteria	0.944 (0.872–1.000)		89 (72–96)	100 (81–100)
2018 Lake Louise criteria excluding mapping	0.870 (0.763–0.978)		73 (55–87)	100 (81–100)
Non-contrast score (native T1 + T2 mapping)	0.920 (0.792–0.982)		85 (64–96)	100 (79–100)

Data are given as percentages with 95% confidence intervals. *AUC* area under the curve

^a^Mapping parameters were available in 26/43 patients and in all controls

### Cardiac magnetic resonance imaging follow-up

**Fig. 3:**
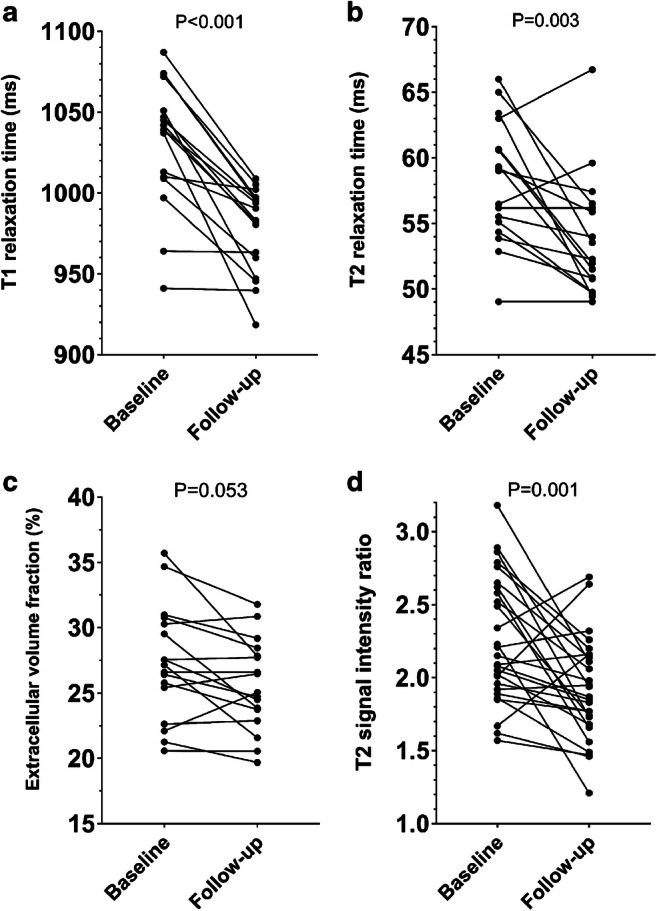
Line graphs show the chronological course of T1 relaxation time (**a**), T2 relaxation time (**b**), extracellular volume fraction (**c**) and T2 signal intensity ratio (**d**) at baseline and follow-up (follow-up was available in *n*=27; mapping parameters were available in *n*=17). Individual values are represented by the *dots* at baseline and follow-up cardiac magnetic resonance. The connecting *lines* show the tendency of change in quantitative parameters over time. *P*-values were obtained using a paired Student’s *t*-test

**Fig. 4:**
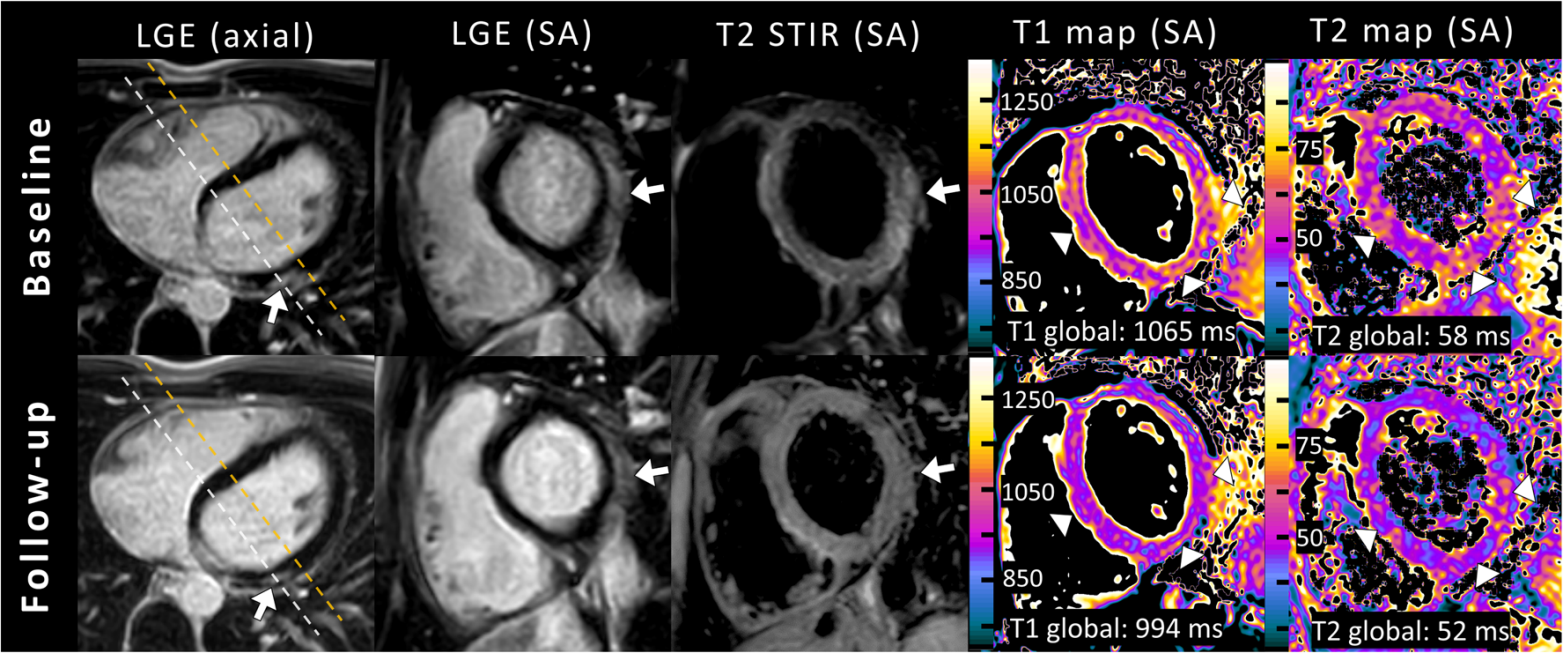
A clinical example of cardiac magnetic resonance imaging (MRI) in a 15-year-old boy with the typical appearance of acute myocarditis on cardiac MRI at baseline and with recovery on follow-up after 2 months. Cardiac MRI in end-diastole shows subepicardial enhancement of the basal lateral wall on late gadolinium enhancement (LGE) images in axial and short-axis (SA) orientation with associated focal myocardial edema (*arrows*) on fat-suppressed (T2-weighted short TI inversion recovery [T2 STIR]) images (*white dashed lines* in axial LGE images represent the imaging plane of LGE and T2 STIR). T2 STIR images at follow-up show normalization of focal myocardial edema. Mapping parameters displayed high myocardial native T1 and T2 relaxation times at baseline cardiac MRI and normalization at follow-up. Notably, in this example, T1 and T2 maps cover the transition between the basal and mid segments outside the visible late gadolinium enhancement lesions (*orange dashed lines* in axial LGE images represent the imaging plane of the quantitative maps) and detected additionally diffuse myocardial alterations in the septal and inferior wall, which normalized at follow-up (*arrowheads*)

**Fig. 5:**
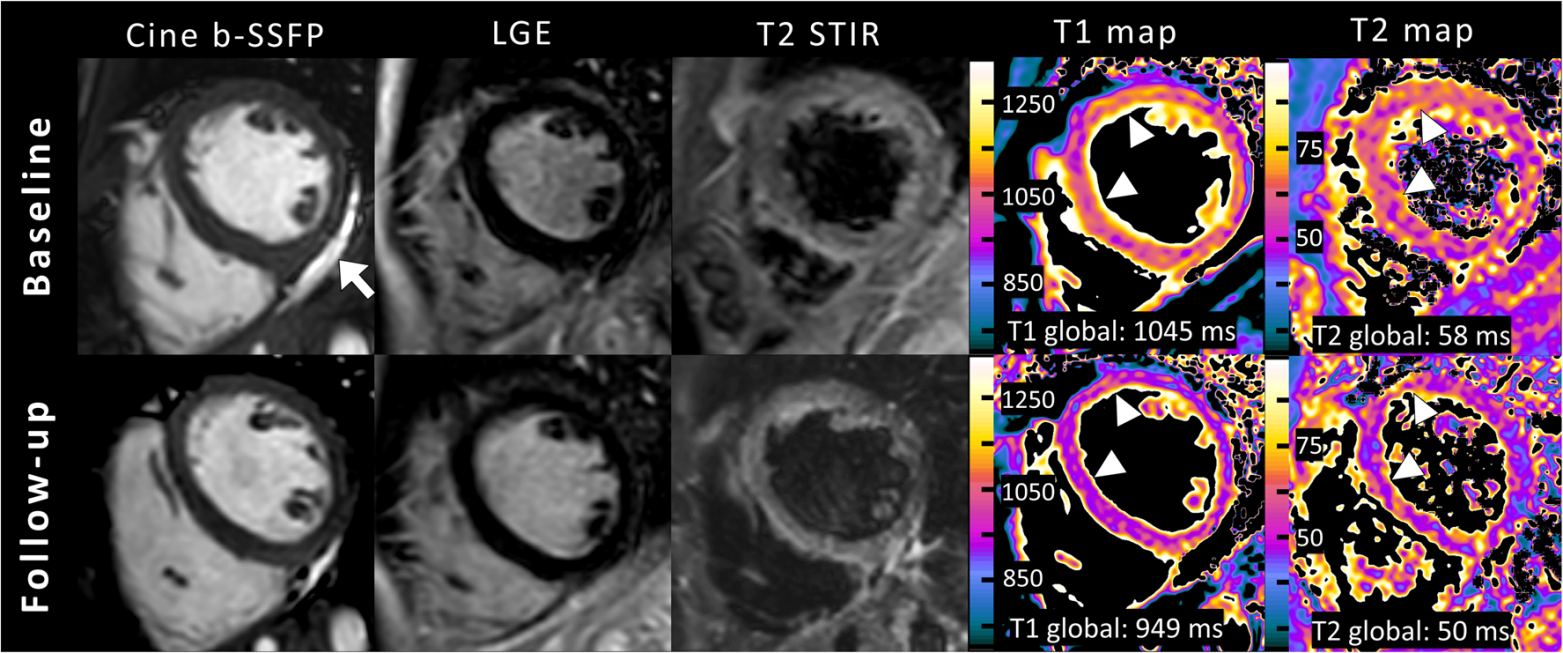
A clinical example of cardiac magnetic resonance imaging (MRI) in short-axis view in a 16-year-old boy. Cine images (balanced steady state free precession [b-SSFP]) show normal left ventricular ejection fraction (LVEF; 58%, no segmental hypokinesia) and pericardial effusion basal inferior (*arrow*). No focal or diffuse enhancement was identified on late gadolinium enhancement (LGE). No focal myocardial edema was visible on fat-suppressed (T2-weighted short TI inversion recovery [T2 STIR]) images. Mapping parameters displayed high global myocardial native T1 and T2 relaxation times at baseline cardiac MRI and normalization at follow-up (*arrowheads* show the most affected segments). The diagnosis of acute diffuse myocarditis in this patient was only possible using quantitative parameters according to 2018 Lake Louise criteria and would have been missed by the original Lake Louise criteria

Cardiac MRI follow-up was performed in 27/43 patients with acute myocarditis. Mapping parameters were available in 17/27 follow-up scans. Median time to cardiac MRI follow-up was 53 days (range: 20–322 days). LVEF improved at follow-up (58±7% vs. 61±4%; *P*=0.039). Visual myocardial edema was initially observed in 17/27 (63%) patients and was still present in 12/27 (44%) patients at follow-up. Late gadolinium enhancement was visible in 25/27 (93%) patients at baseline cardiac MRI and in 20/27 (74%) patients at follow-up. Native T1 (1,032±39 ms vs. 975±33 ms; *P*<0.001) and T2 relaxation times (58±5 ms vs. 54±5 ms; *P*=0.003) decreased at follow-up. Baseline and follow-up parameters are presented in Table 3 and Fig. 3. Representative clinical cardiac MRI examples of patients with acute myocarditis are presented in Fig. 4 (case with a typical pattern of acute myocarditis) and Fig. 5 (case with a diffuse pattern of acute myocarditis, which was diagnosed by mapping parameters according to the 2018 Lake Louise criteria).

**Table 3:** Cardiac magnetic resonance imaging characteristics of pediatric and adolescent patients with acute myocarditis at baseline and follow-up

Variable	Baseline (*n*=27)	Follow-up (*n*=27)	*P*-value
Left ventricular ejection fraction (%)	58±7	61±4	0.039
Left ventricular end-diastolic volume index (mL/m^2^)	83±14	82±15	0.53
Cardiac index (L/min/m^2^)	3.4±0.5	3.4±0.6	1.0
Interventricular septal thickness (mm)	8.6±1.3	8.5±1.3	0.14
T2 signal intensity ratio	2.25±0.43	1.91±0.35	0.001
Visual myocardial edema	17 (63%)	12 (44%)	0.016
Visual late gadolinium enhancement	25 (93%)	20 (74%)	0.063
T1 relaxation time, native (ms)	1,032±39	975±33	<0.001
Extracellular volume fraction (%)	27.9±5.9	25.6±3.5	0.053
T2 relaxation time (ms)	58±5	54±5	0.003

Continuous variables are given as mean±standard deviation. Dichotomous variables are given as absolute frequency with percentages in parentheses. *P*-values were obtained using a paired Student’s *t*-test or McNemar’s test. T1 and T2 relaxation times and extracellular volume fraction were available in 17/27 patients

## Discussion

In this study, we evaluated the diagnostic performance of current cardiac MRI criteria for acute myocarditis in pediatric and adolescent patients. The main finding is that the 2018 Lake Louise criteria have a high performance for the diagnosis of acute myocarditis in pediatric and adolescent patients, especially when mapping parameters are implemented. Monitoring of disease/follow-up is feasible with cardiac MRI, especially with mapping parameters. Extensive increase of T2 relaxation times is associated with a severe course of disease. Furthermore, a combined non-contrast score solely based on native mapping parameters yielded a diagnostic accuracy comparable to the 2018 Lake Louise criteria. Therefore, the use of a non-contrast cardiac MRI protocol in children may be a viable option in cases where intravenous contrast is sought to be avoided.

Several studies have investigated the diagnostic performance of myocardial T1 and T2 mapping and extracellular volume fraction in adults [14, 21–24]. However, cardiac MRI studies evaluating pediatric myocarditis are underrepresented. The published cutoff values for mapping parameters in acute myocarditis show a wide heterogeneity because myocardial T1 and T2 relaxation times depend on different center-specific technical parameters as well as on the employed scanning sequence, field strength, manufacturer, software and type of analysis [25]. Furthermore, physiological factors may influence T1 and T2 relaxation times, e.g., sex, age, heart rate or even hydration status [26–29]. Previous studies have shown an age-related impact on myocardial T1 relaxation times. Rosmini et al. [30] found that native T1 relaxation times were slightly lower with increasing age. Plausible reasons might be myocardial lipofuscin or hemosiderin accumulation. Roy et al. [28] described higher T1 and extracellular volume fraction values in male participants of increased age, probably related to diffuse myocardial fibrosis. The determined cutoff values for the diagnosis of acute myocarditis in children and adolescents in our study differ from cutoff values for adults, which were determined in a previous study by applying the same mapping sequences and overall technical parameters [15], which might imply that appropriate cutoff values are needed for specific patient cohorts.

### 2018 Lake Louise criteria

In a previous adult study, the 2018 Lake Louise criteria (sensitivity: 87.5%, specificity: 96.2%) outperformed the original 2009 Lake Louise criteria (sensitivity: 72.5%, specificity: 96.2%) for diagnosis of acute myocarditis [16]. In a pediatric cardiac MRI study by Cornicelli et al. [31], only 57% of patients met the original 2009 Lake Louise criteria, but the additional use of mapping parameters yielded a higher diagnostic performance. In particular, a combined score of global native T1 and T2 relaxation times yielded a sensitivity of 83% and a specificity of 96% [31]. However, the appropriate application of the 2018 Lake Louise criteria could not be done due to its later release. In our study, we also could confirm high diagnostic accuracy for 2018 Lake Louise criteria for a pediatric and adolescent cohort, which was significantly higher when mapping parameters were implemented into the score (sensitivity: 89% vs. 73%, specificity: 100% vs. 100%).

### T1 mapping

T1 mapping as a single parameter achieved high diagnostic values according to a recent meta-analysis in adults with acute myocarditis (pooled sensitivity: 85%, pooled specificity: 86%) [32]. High sensitivity (91%) and specificity (86%) of T1 mapping were also observed in the reported pediatric cohort by Cornicelli et al. [31]. We could also show a comparable diagnostic performance of T1 mapping with an AUC of 0.873 and a slightly lower sensitivity (sensitivity: 82%, specificity: 100%) in our cohort. These results show that myocardial T1 mapping is a valuable tool for detecting acute myocarditis in a pediatric cohort. An increase of myocardial T1 values is mainly driven by intracellular or extracellular edema, hyperemia and necrosis in acute myocarditis. However, it is also increased in systemic or chronic diseases related to protein deposition, e.g., in diffuse fibrosis causes like chronic myocarditis or ischemic and nonischemic cardiomyopathies [33, 34]. Therefore, myocardial T1 mapping as a single parameter is not specific for acute myocarditis and should always be interpreted in clinical context.

### Extracellular volume fraction

Extracellular volume fraction was implemented as another T1-based criterion in the 2018 Lake Louise criteria [13]. It reflects the volume of cell-free heart tissue, including the intracapillary plasma volume, which is increased in acute myocarditis [33]. However, it also includes the space occupied by the extracellular matrix, and is therefore a surrogate for myocardial fibrosis [32, 33]. In our study, the diagnostic performance of extracellular volume fraction (AUC: 0.687, sensitivity: 58%, specificity: 88%) was lower compared to T1 or T2 mapping, which differs from the pediatric study of Cornicelli et al. [31]. However, a recent meta-analysis also showed lower overall performance of extracellular volume fraction compared to native T1 and T2 mapping [18]. Furthermore, previous studies could demonstrate a strong correlation between extracellular volume fraction values and histological myocardial fibrosis [33, 35]. Thus, extracellular volume fraction could still be normal in the early stage of disease and probably increase in severe or chronic courses of myocarditis.

### T2 mapping

While neither extracellular volume fraction nor native T1 mapping strictly represent acute myocardial injury and are also biomarkers of myocardial fibrosis, T2 mapping has shown high sensitivity for detecting myocardial edema (e.g., inflammatory or ischemic causes) and is considered specific for acute myocardial diseases [20, 22]. Therefore, applying T2 mapping as a parameter of myocardial edema can help to discriminate between acute and convalescent myocarditis and also between chronic myocarditis and noninflammatory dilated cardiomyopathy [22, 36, 37]. For this reason, it was implemented in the 2018 Lake Louise criteria as a T2-based criterion [13]. In our study, T2 relaxation times reached the highest sensitivity (88%) of all single parameters and showed the highest diagnostic performance of all quantitative markers (AUC: 0.899), which is in line with the results of previous adult and pediatric studies [15, 16, 31, 32]. Although T2 mapping is less dependent on field strength compared to T1 mapping, most of the above-mentioned technical and physiological limitations also apply to T2 mapping.

### Non-contrast score

According to the 2018 Lake Louise criteria, the diagnosis of acute myocarditis can be principally based on native parameters only (increased native T1 and T2 relaxation times). In our cohort, a combined native score of T1 and T2 mapping showed a high diagnostic performance (AUC: 0.920, sensitivity: 85%, specificity: 100%), which was comparable to the original 2018 Lake Louise criteria (AUC: 0.944, sensitivity: 89%, specificity: 100%). Although complications regarding gadolinium-based contrast agents are rare, especially since the introduction of new-generation agents, the application has several drawbacks. Peripheral intravenous insertion in children is often a traumatic procedure and extravasation may occur. Furthermore, anaphylactic reaction and nephrogenic systemic fibrosis in end-stage renal disease are extremely rare but serious complications [38]. Given the recent literature on gadolinium retention within the brain and body of adults and children after repetitive contrast-enhanced MRI scans, long-term effects and clinical implications remain unclear [38]. Therefore, guidelines recommend cautious use of gadolinium contrast agents in children. Our results indicate that a non-contrast cardiac MRI protocol is a viable option for the diagnosis of acute myocarditis in cases where intravenous contrast is sought to be avoided. Furthermore, our follow-up results indicate that a non-contrast score can sufficiently track disease activity or therapeutic response. But late gadolinium enhancement imaging remains an essential part of diagnostics in acute myocarditis, especially for detecting focal myocarditis (which may be missed by native quantitative mapping parameters), for discriminating between ischemic and nonischemic cardiomyopathies as well as risk stratification based on visualization and quantification of myocardial scar formation [39–41].

### Clinical correlations

Because the clinical manifestation of myocarditis varies with broad spectrums of symptoms ranging from asymptomatic courses to fulminant presentations with cardiogenic shock, the diagnosis of myocarditis might be difficult. Therefore, in clinical routine, myocarditis is often diagnosed using a multimodal approach with clinical, laboratory, imaging-based and pathological parameters. Our results show correlations between quantitative parameters and disease severity. T1 and T2 values were higher in patients with reduced LVEF. Patients requiring intermediate care unit treatment presented with higher T1 and T2 relaxation times, but without significant differences in extracellular volume fraction, LVEF or troponin level, when compared to patients not requiring intermediate care unit treatment. Notably, T2 relaxation times were directly associated with the duration of intermediate care unit treatment and the extent of systemic inflammation (C-reactive protein, white blood cell count). These findings indicate the diagnostic value of mapping parameters regarding the detection of severe courses of myocarditis, which are associated with increased mortality [42]. Furthermore, quantitative parameters have been shown to be appropriate markers to discriminate between active inflammation and the convalescent stage of the disease [22]. Our results also show that markers of active inflammation (T1 and T2 relaxation times, T2 signal intensity ratio) decreased at follow-up. However, visual myocardial edema and late gadolinium enhancement were still present in most patients. Therefore, mapping parameters seem to be a valuable tool to track disease activity or therapeutic response.

### Limitations

Our study has some limitations. First, endomyocardial biopsy was not available as a reference standard because it is not routinely used in the diagnostic work-up of acute myocarditis at our institution due to relevant disadvantages, e.g., sampling error leading to false-negative results in focal myocarditis or its periprocedural risks, especially in sick children [43, 44]. Therefore, clinical evidence for myocarditis was the reference standard used in this study, which has some drawbacks because this might have influenced the patient composition. Second, most included patients showed an infarct-like pattern of myocarditis. However, cutoff values might be different in patients with more chronic symptoms. Third, clinical data are partially incomplete because of the retrospective study design. Fourth, infants and young children are underrepresented in our cohort; however, the overall incidence rate of acute myocarditis is very low in this age group. Fifth, our single-center cohort is small, therefore additional prospective studies are needed before our results can be generalized.

## Conclusion

The 2018 Lake Louise criteria including mapping parameters have a high performance for the diagnosis of acute myocarditis in children and adolescents and should be implemented into routine diagnostic work-up. Mapping parameters might be especially useful in tracking inflammatory disease activity.

## Supplementary Information

Online Supplementary Material 1 (DOCX 20 kb)
