# Supplementary material for: Multiparametric cardiac magnetic resonance imaging in pediatric and adolescent patients with acute myocarditis
Source: Pediatr Radiol. 2021 Aug 25;51(13):2470–80. doi: 10.1007/s00247-021-05169-7 (PMC8599260; doi:10.1007/s00247-021-05169-7)
Supplement: Supplementary file 1 — (DOCX 20 kb) [file 247_2021_5169_MOESM1_ESM.docx]

**Appendix S1**

Technical parameters of quantitative cardiac magnetic resonance sequences:

*T1 mapping and ECV*

For myocardial T1 mapping, a standard, electrocardiogram synchronized 3(3)3(3)5 modified Look-Locker inversion recovery (MOLLI) acquisition scheme^17^ was applied using the following parameters: time of repetition 2.2 ms, time of echo 1.02 ms, flip angle 35°, parallel imaging factor 2, acquired voxel size 1.97 x 2 × 10 mm, reconstructed voxel size 1.17 × 1.17 x 10 mm, scan duration/breath hold 15.0 s, 3 slices. Post-contrast T1 maps were performed 10 minutes after contrast injection in the same positions as pre-contrast examinations using the same imaging technique.

*T2 mapping*

For myocardial T2 mapping, a six-echo gradient spin echo (GraSE) sequence^16^ was used with application of the following parameters: time of repetition 1 RR interval, time of echo 23.6/∆TE = 11.8 (6Ec), flip angle 90°, parallel imaging factor 2, acquired voxel size 1.97 × 2.03 × 10 mm, reconstructed voxel size 1.03 × 1.03 x 10 mm, scan duration/breath hold 14.0 s, 3 slices.

**Table S1.** Exemplary sequence parameters of the cardiac magnetic resonance scan protocol.

| **Parameter** | **SSFP Cine (SA)** | **Black-blood T2 STIR (SA)** | **LGE (SA)** |
| --- | --- | --- | --- |
| Field of view (mm) | 350 x 350 | 350 x 350 | 360 x 311 |
| TR (ms) TE (ms) | 2.8 1.38 | 2 RR intervals 70 | 3.5 1.71 |
| Flip angle (°) | 60 | 90 | 15 |
| Voxel size (mm) acquired,  reconstructed | 1.79 x 2 x 8 0.99 x 0.99 x 8 | 1.51 x 2.43 x 8 0.91 x 0.91 x 8 | 1.65 x 1.88 x 10 0.9 x 0.9 x 5 |
| Parallel imaging factor | 3 | 2.5 | 2 |
| Scan duration | 1 min 24 s | 1 min 36 s | 27 s |
| Scan time/ breath-hold | 13 s | 8 s | 12 s |
| Cardiac phases per RR interval Shot duration (ms) | 40  - | -  134 | -  151 |

SSFP=steady-state free precession, STIR=short-tau inversion recovery, LGE=late gadolinium enhancement, SA=short axis view, TR=time of repetition, TE=time to echo.
